# Quantitative analysis of the diffusion characteristics of China's long-term care insurance policy based on the PMC index model

**DOI:** 10.3389/fpubh.2025.1467062

**Published:** 2025-05-22

**Authors:** Peixin Duan, Jiayi Wang, Ruisi Zhang, Haiyan Jia, Hui Sun, Yanfang Li

**Affiliations:** ^1^School of Public Administration and Policy, Shandong University of Finance and Economic, Jinan, China; ^2^Department of Basic Education, Shandong College of Electonic Technology, Jinan, China

**Keywords:** long-term care insurance, policy diffusion, PMC index model, policy pilot, policy evaluation

## Abstract

**Background:**

Policy pilots and policy diffusion are important tools for national governance and policy innovation in China. Long-term care insurance (LTCI) offers a potential solution to the challenges posed by the aging population. Currently, the pilot program for the LTCI system in China has gone through two phases, but a unified national policy framework has yet to be established. Therefore, the outcomes of the policy pilots and the diffusion process of this policy warrant further exploration.

**Method:**

Based on the perspective of policy diffusion, this study employs textual analysis and applies the Policy Modeling Consistency (PMC) index model to evaluate the quality and consistency of LTCI policies. A quantitative evaluation is conducted on 29 LTCI policies from two groups of pilot cities in China. The characteristics of policy diffusion are analyzed from both temporal and spatial dimensions.

**Conclusion:**

(1) According to the PMC index results, LTCI policies of 25 cities achieved an excellent level, 4 cities reached an acceptable level, and there were no substandard policies among the 29 pilot cities. The overall consistency of LTCI policies in the first batch of pilot cities in China is higher than that in the second batch, with PMC index of 6.61 and 6.23. The LTCI policy still faces many challenges, such as limited funding sources and a narrow scope of care services. (2) The diffusion of LTCI policy across different regions and batches has the following characteristics: the diffusion of LTCI policy has followed an “M-shaped” curve over time. In terms of spatial diffusion, there is an interaction between spatial proximity and social proximity effects. In terms of diffusion pathways, there is a combined effect of vertical and horizontal diffusion, reflecting a hierarchical diffusion pattern.

**Discussion:**

This paper constructs a new analytical framework for studying policy diffusion. Based on the analysis of policy texts using the PMC model, we focus on the analysis of policy quality and consistency to the diffusion characteristics and exploring the underlying reasons for the diffusion of the LTCI policies. This extends the research of LTCI policy from traditional qualitative analysis to the quantitative research domain.

## 1 Introduction

By the end of 2023, China's population aged 60 and above had exceeded 296 million, accounting for 21.1% of the total population, with individuals over 65 accounting for 14.9%. This indicates that China has entered the stage of a deeply aging society. Additionally, the number of disabled and semi-disabled older adult continues to rise. According to data from the Ministry of Civil Affairs of China, there were 35 million disabled and semi-disabled older adult people in 2022, and is projected to rise to 58 million by 2050 (1). The escalating demand for the long-term care among disabled older adult individuals has become a pressing issue, posing one of the most critical challenges brought about by the raid aging of the population.

In response to the increase demand for older adult care, China has been actively developing a long-term care insurances (LTCI) system. Qingdao was the first city to introduce a LTCI system in July 2012 as a measure to address growing social tensions (2). Also, the initiative produced positive social outcomes. Following this, the Ministry of Human Resources and Social Security (MHRSS) initiated a pilot program in 2016, selecting 15 cities to trial the LTCI system (3, 4). Subsequently, in 2020, the central government launched the second wave of LTCI pilot projects. This expansion involves the selection of an additional 14 cities from provinces without existing pilot cities (5).

Policy pilot initiatives are a distinctive feature of China's governance system and are essential for understanding its administrative mechanisms. A policy pilot refers to a small-scale policy implementation in a limited geographic area and among targeted population, designed to assess the feasibility and effectiveness of a policy before its broader application. It aims to achieve “point-to-surface” approach and further enables the wider dissemination of successful pilot experiences. The LTCI policy serves as a typical example of this approach, progressing through three stages: the initial pilot stage, the localized pilot stage, and the expanded pilot stage. Notably, the 20th National Congress Report calls for the “establishment of a unified nationwide LTCI policy framework during the 14th Five-Year Plan”.

As the number of LTCI policies increases, the policy system becomes more complex. Despite the widespread promotion of LTCI policies across most regions of China, significant regional disparities persist. The central government has yet to issue a national policy document. What are the specific indicators of these policies' overall quality and individual differences? What are diffusion characteristics from the first to the second batch of LTCI policies? What LTCI policies have been issued at the national level, and what is the long-term trend in policy releases? How can we identify the strengths and weaknesses in policy design and provide targeted optimization suggestions? Therefore, we focus on the analysis of policy quality and consistency to the diffusion characteristics and exploring the underlying reasons for the diffusion of the LTCI policies. We employ the PMC model to conduct a quantitative analysis of policy texts from 29 LTCI pilot cities in China.

The potential contributions of this paper are summarized in three main points. Firstly, this paper constructs a new analytical framework for studying policy diffusion based on the analysis of policy texts using the PMC model. Currently, research on the diffusion of LTCI policies remains relatively limited. While some scholars have examined the characteristics of policy diffusion from the perspectives of time, space, institutional frameworks (6), social networks (7), and the policy tool (8). These researches primarily use qualitative methods, summarizing the diffusion by reviewing the number and content of policies across different regions (9). Using the PMC model, we integrate policy actors, policy tools, and policy processes, constructing a multidimensional evaluation index system to systematically analyze the LTCI. This framework provides a basis for exploring the diffusion characteristics of the policy and offers insight into the distinct pathways and experiences underlying the diffusion of LTCI in China. Secondly, this study conducts an exploratory quantitative evaluation of LTCI policies in China based on the PMC model. Twenty-nine policy documents included in the study were text-mined. Based on the text mining results, the PMC model was constructed from multiple dimensions to assess the quality and consistency of LTCI policies. Thirdly, we focus on the design and formulation process to evaluate LTCI policies. The research about the evaluation of LTCI policies emphasizes policy implementation effects and influencing factors, but fewer studies examine the design and formulation processes of LTCI policies. Despite the widespread promotion of LTCI policies across most regions of China, significant regional disparities persist. The central government has yet to issue a national policy document (10), and the consistency and effectiveness of LTCI policies implementation require improvement. Addressing the consistency of LTCI policy formulation and policy diffusion in this paper is crucial for resolving this issue. Therefore, summarizing the diffusion characteristics and exploring the underlying reasons for the diffusion of the LTCI policies can help deepen the understanding of the localized LTCI system and enrich the theory of public policy innovation and diffusion in China.

The rest of this paper is organized as follows. Section 2 presents the literature review, Section 3 describes the data, the framework and the methods. Section 4 examines the empirical results about PMC model. Section 5 further analyzes diffusion characteristics of LTCI policies based on results of PMC model. Section 6 and Section 7 summarize the results of the study, provides guidance, and discusses policy recommendations.

## 2 Literature review

As the aging population continues to grow, the Chinese government has been actively exploring the establishment of a long-term care security system. Qingdao initially implemented a LTCI policy, which was later expanded to the national level through two pilot programs. Hence, it is an institutional change that was induced “bottom-up” (11). As the pilot LTCI policy progresses, a growing number of studies have endeavored to assessing the LTCI policies in China.

Research on LTCI policies in China generally falls into two main methodological categories: qualitative and quantitative analysis. Quantitative studies primarily focus on assessing the impact of LTCI policies on various economic and social aspects in one or more pilot cities using micro-level data, such as the CHARLS and CLHLS datasets. Quantitative analysis literature primarily focuses on evaluating the effects of LTCI on various economic and social aspects in one or more pilot cities. For example, Na Cao analyzed the implementation of the LTCI system using a fixed effects model and found that the implementation of the LTCI system can reduce disability among the older adult (12). These researches use micro-level data, such as CHARLS and CLHLS dataset to study examines the impact of LTCI on older adult health (13, 14), medical expenses (15, 16) and the use of health services (17, 18). The difference-in-differences (DID) model is used to differentiate and analyze the policy effects (19–22). This method has been widely employed by scholars in LTCI policy research to evaluate the effects of policy implementation (16, 23–25). Qualitative analysis primarily focuses on review the key characteristics of LTCI in different countries and areas (9, 26, 27).

The evaluation of LTCI policies is complex and most scholars concentrate on the effects of policy implementation (16, 23–25) while giving less attention to individual differences and the texts of policies. Especially, there is a lack of systematic research on LTCI policy design and texts. The PMC model combines qualitative and quantitative approaches in a more comprehensive and objective way than the above methods, which are widely used to evaluate policies (22, 28). It can provide an overall evaluation of policy consistency as well as systematically analyze individual policy differences from various dimensions (29). At present, the PMC model is mostly used by scholars in the fields of public administration, social security and social organizations scientific research and technical services, *etc*. (30). However, the LTCI policy has not yet been established as a national framework, and different localities in China are still exploring its implementation. Therefore, it is important to analyze not only the policy text of LTCI policies but also its diffusion characteristics support the development of a unified national framework.

Based on Rogers' “diffusion of innovations” theory (31) policy diffusion refers to the phenomenon where a government's adoption of a particular policy is influenced by the choices of other governments (32). This process can occur through multiple mechanisms, including coercion (where policy choices are shaped by incentives or penalties imposed by higher authorities), learning (where decision-makers evaluate the effectiveness of policies previously adopted elsewhere), and competition (where governments adopt similar policies to gain comparative advantages) (33). Rogers proposed that the innovation diffusion process unfolds in five stages: knowledge, persuasion, decision, implementation, and confirmation. His research on regional diffusion patterns revealed that the rate of innovation adoption typically follows an S-shaped curve, assuming a natural diffusion trajectory (31). However, LTCI policy pilot program exhibits distinct “central government-led, local government-responsive” characteristics (34). The PMC model addresses this contextual specificity by incorporating multi-dimensional indicators to capture hierarchical and regional variations in policy design, thereby overcoming the limitations of traditional models in explaining China's unique policy diffusion patterns.

This paper uses a text analysis method to construct the PMC model to examine the characteristics of policy diffusion. We analyze the characteristics of LTCI policies across the dimensions of policy nature and insured objects, quantitatively assess policy formulation and hierarchy, and explore the diffusion characteristics of LTCI policies based on the PMC model's quantitative results. By analyzing the PMC evaluation indicators, the study explores the diffusion characteristics of the two batches of pilot policies and investigates the pathway and experience unique to China's LTCI policy diffusion process.

## 3 Research design

### 3.1 Data collection

As of 2023, 29 cities have been included in the national pilot program for LTCI in China and we select all these cities as samples for analysis. To ensure the accuracy and comprehensiveness of the research data, the sample selection process followed these steps: (1) Keywords such as “long-term care insurance,” “long-term care,” “long-term nursing” and “long-term disability” were used to collect relevant policy documents from sources including the Peking University Law and Regulation Database, the National Healthcare Security Administration, the National Health Commission, the Ministry of Human Resources and Social Security, as well as provincial and municipal healthcare security bureaus and local governments. A total of 1,351 policy documents were collected from 29 pilot regions between 2012 and 2023. (2) Screening of policy texts was conducted based on completeness, relevance, timeliness and operability. Due to incompleteness, outdated information, or limited relevance to LTCI, 812 policies were excluded. (3) One policy was selected from each pilot city as a research sample based on relevance, authority, and representativeness. Ultimately, 29 policies were selected as research subjects (Table 1).

**Table 1:** Policy texts of two batches of LTCI pilot areas.

**Number**	**Policy name**	**Year**
P1	Notice on further advancing the implementation of LTCI pilot work	2022
P2	Implementation rules of LTCI in Chengdu City	2022
P3	Trial measures for LTCI system in Kaifeng City	2021
P4	Comprehensive implementation plan for LTCI system	2019
P5	Opinions on expanding the implementation of LTCI pilot system	2021
P6	Measures for LTCI in Jingmen City (Trial)	2019
P7	The interim measures for LTCI in Qingdao	2018
P8	Opinions on establishing a basic care insurance system (Trial)	2015
P9	Notice on issuing the pilot program for LTCI in Ningbo City	2017
P10	Pilot program for LTCI system in Shijingshan district (trial)	2018
P11	Pilot implementation plan for LTCI system in Hohhot City	2020
P12	Trial measures for LTCI pilot in Shanghai	2021
P13	Issued the comprehensive pilot program for LTCI system	2021
P14	Implementation rules for LTCI system in Xiangtan City (Trial)	2021
P15	Management measures for LTCI for urban employees in Chengde	2021
P16	Pilot implementation plan for deepening the LTCI system in Qiqiha	2021
P17	Jincheng LTCI related work system notice	2021
P18	Trial implementation rules for LTCI in Qiannan prefecture	2020
P19	Opinions on the implementation of LTCI pilot system in Nanning City	2021
P20	Notice on issuing the implementation rules for LTCI in Shihezi City	2017
P21	Notice for further advancing the pilot program of LTCI in Tianjin	2022
P22	Implementation rules for LTCI in Fuzhou City	2021
P23	Implementation plan for LTCI system in Panjin	2020
P24	Implementation rules for LTCI in Urumqi City	2022
P25	Implementation measures for LTCIe for urban employees in anqing	2020
P26	Implementation measures for LTCI in Hanzhong City (Trial)	2020
P27	Opinions on establishing medical care insurance system for disabled	2015
P28	Trial measures for LTCI in Guangzhou City	2020
P29	Implementation rules for LTCI for employees in Gannan Prefecture	2021

### 3.2 Research framework

**Figure 1:**
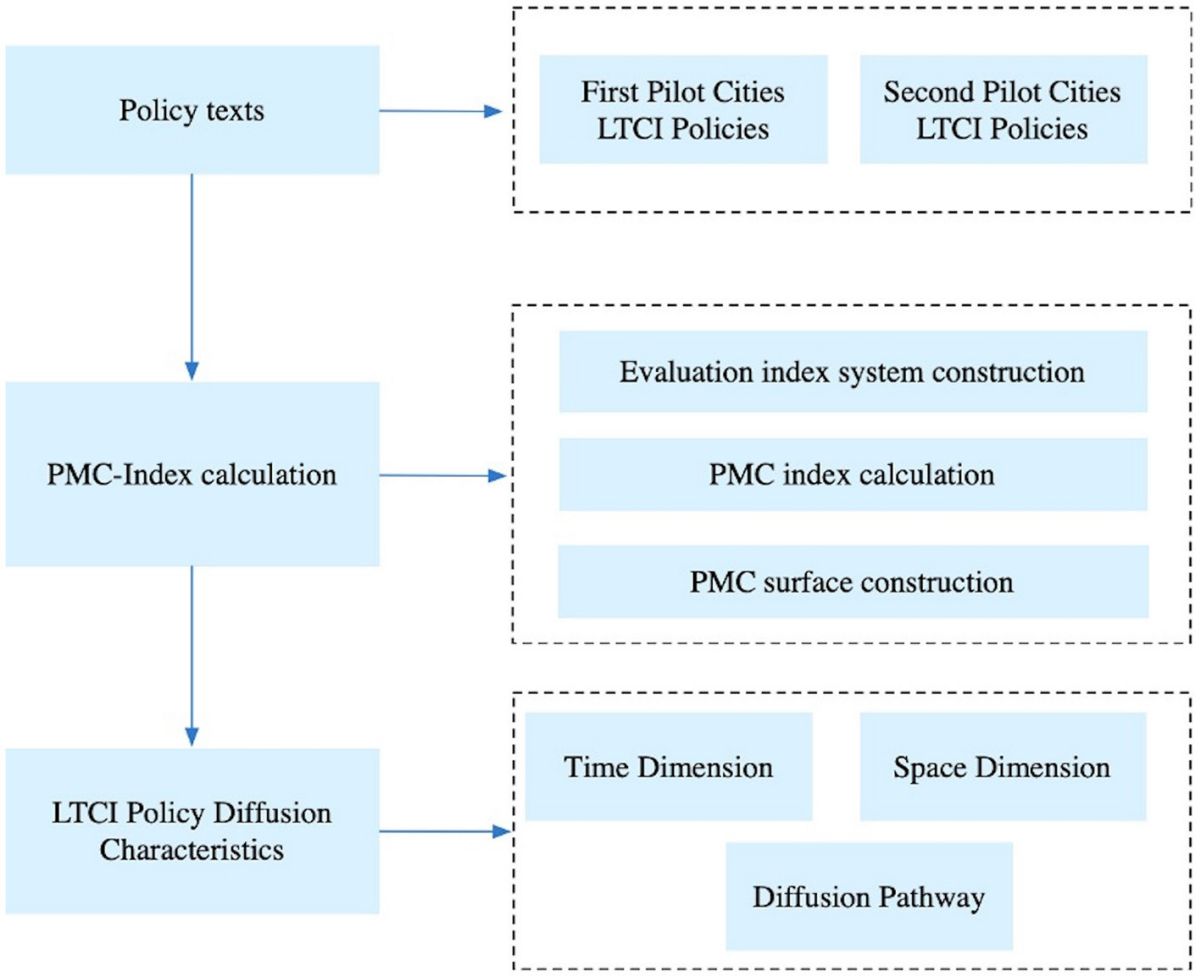
LTCI framework for analyzing policy diffusion.

The central government has yet to issue a national policy document in China, and the consistency and effectiveness of LTCI policies implementation require improvement. We focus on the consistency of LTCI policy formulation and policy diffusion in this paper. So we use the PMC model to conduct a quantitative analysis of policy texts from 29 LTCI pilot cities to examine the diffusion characteristics. The specific analytical framework is shown in Figure 1.

First, we collect all LTCI policies published in the two batches of pilot regions in China from 2012 to 2023, and then screens the collected these policies to select one representative policy from each pilot region as the research sample. Second, we use the PMC model to conduct a quantitative analysis of policy texts from multiple perspectives. The PMC model can provide an overall evaluation of policy consistency as well as systematically analyze policy differences from various dimensions. Finally, based on the analysis of the PMC results we conducted a study of the characteristics of policy diffusion from the dimensions of time, space, and pathway.

### 3.3 Research methodology

#### 3.3.1 Concept of PMC index model

The PMC index model was first proposed by Estrada (35) as a quantitative evaluation framework for individual policies. The model is based on the idea that all things are dynamic and interconnected, and that every variable is crucial (36). Therefore, comprehensive consideration is needed when evaluating indicator systems. It has since become a key method for quantitative research in policy literature. The model constructs an indicator system to assess the internal consistency of a single policy, reflecting both the overall policy and its specific aspects.

#### 3.3.2 PMC index model construction

The PMC index model follows five basic steps: (1) classifying variables and identifying parameters; (2) establishing a multi-input–output table; (3) calculating the PMC index; (4) constructing the PMC surface diagram; (5) conducting a comprehensive analysis of policy strengths and weaknesses based on specific criteria.

(1) Classifying variables and identifying parameters. First, an evaluation indicator system was constructed based on the LTCI policy texts, including both primary and secondary indicators X~N[0,1]. According to the PMC index model construction, all relevant and potential variables should be considered when establishing the evaluation system for LTCI policies. This paper, based on previous research (29, 30, 37, 38) and the specific characteristics of LTCI policy texts, establishes nine primary indicators, as detailed below: Policy Nature (X1), Policy Function (X2), Policy Prescription (X3), Insured Object (X4), Capital Source (X5), Policy Norm(X6), Nursing Service (X7), Service Place (X8), and Safeguard Measure (X9).

These indicators are established within the framework of policy diffusion theory (32, 39) and social network theory (40), aiming to analyze the diffusion characteristics across two batches of policy pilot projects. Policy Nature (X1) explain how the two batches of pilot policies reflect the initial diffusion stage of LTCI policy, as well as the differing operational approaches of local governments during the pilot phase. Policy Function (X2) refers to the requirement from central authorities for local governments to implement “categorized supervision” and “clear delineation of responsibilities” in the context of Long-Term Care Insurance (LTCI) policy. This indicator assesses whether such directives are adequately reflected in local policy documents and whether variations exist across different pilot batches. Policy Prescription (X3) captures policy timeliness, with a focus on evaluating the sustainability of the two LTCI policy batches. Insured Object (X4) focuses on the insured population, investigating whether there are demographic differences between the two policy batches. Capital Source (X5) addresses funding sources, highlighting the crucial role of financial support in policy implementation, particularly how local economic conditions influence the diffusion and execution of the policy. Policy Norm (X6) and Nursing Service (X7) explains the service content, including medical care, rehabilitation, and psychological counseling, emphasizing the importance of policy quality and consistency. Differences in regional policy coverage of sub-items such as “medical care” and “psychological counseling” reflect regional disparities in policy service capacity. Service Place (X8) focuses on service venues, analyzing whether neighboring cities share infrastructure, leading to convergence in policy design. Finally, Safeguard Measure (X9) refers to guarantees, where central documents emphasize “dynamic management” and “information sharing,” requiring local policies to implement specific measures.

Second, based on a content analysis of policy texts and referencing related studies by scholars such as Zhang and Geng (37), Ba et al. (41), and Xiao et al. (42). Forty secondary indicators were set under these nine primary indicators, forming the evaluation system for China's LTCI policies (Tables 2, 3).

**Table 2:** Evaluation index system and standards for LTCI policies (Part I).

**Primary variables**	**Secondary variables**	**Evaluation standards**	**Yes**	**No**
*X*_1_-policy nature	*X*_11_- description	Describe the nature of LTCI.	1	0
	*X*_12_-suggestion	To provide guidance on LTCI system.	1	0
	*X*_13_-supported	Support the work of LTCI system from the perspective of policy coordination, organization and management.	1	0
	*X*_14_-oriented	From the perspective of policy subject and policy recipient, the LTCI system should be emphasized and guided.	1	0
*X*_2_-policy function	*X*_21_-clarify authority and responsibility	Clarify the rights and responsibilities of each party.	1	0
	*X*_22_-specification boot	Policy guidance norm.	1	0
	*X*_23_-classification regulation	Reflecting the idea of classification supervision.		
	*X*_24_-coordination	To promote the pilot project as a whole and serv as an institutional signal reflecting cross-sectoral collaboration between LTCI and healthcare or pension systems.	1	0
*X*_3_-policy prescription	*X*_31_-short term	The validity period is within 2 years (inclusive).	1	0
	*X*_32_-metaphase	Valid for 2–5 years (inclusive).	1	0
	*X*_33_-long term	The validity period is more than 5 years.	1	0
*X*_4_-insured object	*X*_41_-urban workers	Coverage of urban workers.	1	0
	*X*_42_-urban and rural residents	Coverage of urban and rural residents.	1	0

**Table 3:** Evaluation index system and standards for LTCI policies (Part II).

**Primary variables**	**Secondary variables**	**Evaluation standards**	**Yes**	**No**
*X*_5_-policy function	*X*_51_-medical insurance pooling fund	The source of funding includes the Medical Insurance Pooling Fund to component demonstrates the level of integration between LTCI and basic medical insurance schemes.	1	0
	*X*_52_-personal account	The source of funding includes an individual account in Medicare.	1	0
	*X*_53_-individual payment	The source of funds includes additional personal contributions.	1	0
	*X*_54_-unit payment	The source of funds includes the unit's additional contribution or the unit's supplementary contribution.	1	0
	*X*_55_-financial subsidies	The source of funds includes government financial subsidies.	1	0
	*X*_56_-welfare fund	The source of funds includes the welfare fund.	1	0
*X*_6_-policy function	*X*_61_-funding criteria	There are clear funding criteria.	1	0
	*X*_62_-funding principles	The adoption of the “revenue-determined expenditure” funding principle.	1	0
	*X*_63_-responsibility sharing	Multiple parties share the payment responsibility.	1	0
	*X*_64_-disability assessment	There is a clear assessment criteria.	1	0
	*X*_65_-welfare eligibility	If the older adult with dementia are considered as the object of protection.	1	0
*X*_7_-policy function	*X*_71_-medical care	Providing medical care to policy recipients to evaluate whether pilot cities have established clear policy pathways for healthcare resource integration.	1	0
	*X*_72_-daily living care	To provide daily living care for policy recipients.	1	0
	*X*_73_-preventive care	Preventive care for policy recipients.	1	0
	*X*_74_-rehabilitation therapy	Rehabilitation treatment for policy recipients was indicator captures the systemic coordination between LTCI and medical services.	1	0
	*X*_75_-psychological counseling	To provide psychological counseling to policy recipients.	1	0
	*X*_76_-hospice care	To provide hospice care to policy recipients.	1	0
*X*_8_-service place	*X*_81_-hospital	The place of providing service to the policy recipient is hospital.	1	0
	*X*_82_-nursing home	The number of places providing services to policy recipients includes older adult care institutions.	1	0
	*X*_83_-community service center	The service locations for policy recipients are community service centers.	1	0
*X*_9_-safeguard measure	*X*_91_-financial security	Transfer of start-up capital/establishment of adjustment fund/provision of risk reserve.	1	0
	*X*_92_-laws and regulations	Provide legal specifications.	1	0
	*X*_93_-organizational leadership	Coordinate organizational leadership.	1	0
	*X*_94_-fund management	Clarify the fund management method.	1	0
	*X*_95_-information management	Use of “internet plus” to promote LTCI information sharing to reflects local governments' capacity for interdepartmental data sharing and collaborative governance.	1	0
	*X*_96_-dynamic management	In the process of implementing the LTCI system, all localities implement dynamic management.	1	0

(2) Establish a multi-input–output table, and according to the LTCI policy text, second-level variables are assigned a value of 0 or 1. If the content of the policy involves the variable, the content is assigned the value of 1; otherwise, 0, as shown in Equation 1.


(1) $$\begin{array}{l} X=\left\{XR:\left[0,1\right]\right\}. \end{array}$$

(3) Calculating the first-level variable. Calculate the value of the first-level variable, according to the second-level variable parameters, to take the value of the cumulative, as shown in Equation 2.


(2) $$\begin{array}{l} X_{T}=\overset{N}{\underset{J=1}{\sum }}\frac{X_{tj}}{T\left(X_{t}\right)},\;t=1,2,3...... \end{array}$$

where *t* represents the first-level variable, *j* represents the second-level variable, and *T*(*X*_*t*_) represents the number of second-level variables within the first-level variable.

(4) To calculate the final PMC index for a given policy, the fiest-level variables derived in the previous step are summed up and calculated as in Equation 3.


(3) $$\begin{array}{l} \text{PMC}=X_{1}\left(\overset{4}{\underset{j=1}{\sum }}\frac{X_{1j}}{4}\right)+X_{2}\left(\overset{4}{\underset{j=1}{\sum }}\frac{X_{2j}}{4}\right)+X_{3}\left(\overset{3}{\underset{j=1}{\sum }}\frac{X_{3j}}{3}\right)\\ +X_{4}\left(\overset{2}{\underset{j=1}{\sum }}\frac{X_{4j}}{2}\right)+X_{5}\left(\overset{6}{\underset{j=1}{\sum }}\frac{X_{5j}}{6}\right)+X_{6}\left(\overset{5}{\underset{j=1}{\sum }}\frac{X_{6j}}{5}\right)\\ +X_{7}\left(\overset{6}{\underset{j=1}{\sum }}\frac{X_{7j}}{6}\right)+X_{8}\left(\overset{3}{\underset{j=1}{\sum }}\frac{X_{8j}}{3}\right)+X_{9}\left(\overset{6}{\underset{j=1}{\sum }}\frac{X_{9j}}{6}\right). \end{array}$$

(5) Constructing the PMC surface diagram. To more intuitively reflect the strengths and limitations of each LTCI policy. This paper transformed the PMC surface diagram to a 3 × 3 matrix from the specific values of nine primary variables. This diagram is drawn by MATLAB. The calculation results are shown in Equation 4.


(4) $$\begin{array}{l} \text{PMC}\left(\text{meshes}\right)=\left[\begin{array}{ccc} P1 & P2 & P3\\ P4 & P5 & P6\\ P7 & P8 & P9 \end{array}\right]. \end{array}$$

## 4 Results of PMC model

### 4.1 Holistic calculation results evaluation of PMC index

The PMC index and ranking of each sample are calculated using the PMC model (Table 4). These specific values and rankings effectively reveal the overall quality level of the samples. Next, this paper will perform an overall analysis and typical case analysis.

**Table 4:** PMC index scores and rankings of 29 LTCI pilot policies.

**Batch**	**Number**	***X*_1_**	***X*_2_**	***X*_3_**	***X*_4_**	***X*_5_**	***X*_6_**	***X*_7_**	***X*_8_**	***X*_9_**	**PMC index**	**Rank**	**Grade**
1	P7	1	1	0.33	1	0.5	1	1	1	1	7.83	1	Excellent
1	P6	1	1	0.33	1	0.5	0.8	1	1	0.67	7.3	2	Excellent
1	P16	1	1	0.33	0.5	0.83	0.8	1	1	0.83	7.29	3	Excellent
1	P1	0.75	1	0.33	1	0.5	1	0.67	1	0.83	7.08	4	Excellent
1	P12	1	1	0.33	1	0.5	0.8	0.33	1	1	6.96	5	Excellent
1	P9	1	1	0.33	0.5	0.8	0.8	0.33	1	1	6.76	6	Excellent
1	P8	0.5	1	0.33	0.5	0.83	1	0.83	1	0.67	6.66	7	Excellent
1	P4	0.75	1	0.33	1	0.67	1	0.33	0.67	0.83	6.58	8	Excellent
1	P5	0.75	1	0.33	0.5	0.67	0.8	0.67	1	0.83	6.55	9	Excellent
1	P2	0.5	1	0.33	1	0.67	0.6	0.83	0.67	0.83	6.43	10	Excellent
1	P28	0.5	1	0.33	1	0.5	0.6	0.83	1	0.67	6.43	11	Excellent
1	P25	0.75	1	0.33	0.5	0.5	0.8	0.5	1	1	6.38	12	Excellent
1	P20	0.75	1	0.33	1	0.67	0.8	0.33	0.67	0.5	6.05	13	Excellent
1	P15	0.75	1	0.33	0.5	0.5	0.8	0.33	1	0.5	5.71	14	Acceptable
1	P27	0.5	1	0.33	0.5	0.5	0.8	0.33	0.67	0.5	5.13	15	Acceptable
The first batch of sub-means		0.77	1	0.33	0.77	0.61	0.83	0.62	0.91	0.78	6.61		Excellent
2	P3	1	1	0.33	1	0.5	0.8	0.67	0.67	0.83	6.8	16	Excellent
2	P13	0.75	1	0.33	1	0.67	0.8	0.5	0.67	0.83	6.55	17	Excellent
2	P19	0.75	1	0.33	1	0.67	0.8	0.5	0.67	0.83	6.55	18	Excellent
2	P24	1	1	0.33	1	0.67	0.8	0.33	0.67	0.67	6.47	19	Excellent
2	P18	0.75	1	0.33	0.5	0.67	0.8	0.83	0.67	0.83	6.38	20	Excellent
2	P29	0.75	1	0.33	0.5	0.67	0.8	0.33	1	1	6.38	21	Excellent
2	P10	0	1	0.33	1	0.5	0.8	1	1	0.67	6.3	22	Excellent
2	P11	0.5	1	0.33	1	0.67	0.8	0.33	1	0.67	6.3	23	Excellent
2	P14	1	1	0.33	0.5	0.5	0.8	0.33	0.67	1	6.13	24	Excellent
2	P23	0.75	1	0.33	0.5	0.67	0.8	0.33	1	0.67	6.05	25	Excellent
2	P26	0.75	1	0.33	0.5	0.5	0.8	0.33	1	0.83	6.04	26	Excellent
2	P17	0.75	1	0.33	0.5	0.5	0.6	0.33	1	1	6.01	27	Excellent
2	P21	0.75	1	0.33	0.5	0.67	0.6	0.33	1	0.67	5.85	28	Acceptable
2	P22	0.75	1	0.33	0.5	0.5	0.6	0.33	0.67	0.67	5.35	29	Acceptable
The second batch of sub-means		0.73	1	0.33	0.71	0.6	0.76	0.46	0.86	0.8	6.23		Excellent

Samples with a PMC index score between [8, 9] are classified as “Perfect,” those with scores between [6, 7.99] as “Excellent,” scores between [4, 5.99] as “Acceptable,” and scores between [0, 3.99] as “Poor.” Calculation of the PMC index for each pilot city reveals that the average PMC score for the first batch of pilot cities is higher than that of the second batch. None of the LTCI policies issued in the pilot cities from either batch reached the “Perfect” level. Of the 29 sample cities, 25 achieved the “Excellent” level, with 52% from the first batch and 48% from the second batch. Four cities reached the “Acceptable” level, with two cities from each batch classified as such. In the first batch, the PMC index difference between the highest-ranked pilot city (P7) and the lowest-ranked city (P27) is 2.7. In the second batch, the PMC index difference between the highest-ranked pilot city (P3) and the lowest-ranked city (P22) is 1.45. Overall, it can be observed that the LTCI policies from the first batch of pilot cities are superior to those of the second batch. However, the PMC scores among the second batch of pilot cities are more evenly distributed, with smaller differences between cities.

The largest difference between the X7 scores of the first and second pilot sites was 0.16. Analyzing the secondary indicators, we found that the first pilot sites scored significantly higher than the second pilot sites on X73, X74, X75, and X76. The second pilot cities mainly provide medical care and daily living care for care people. The first pilot cities will provide preventive care, rehabilitation therapy, psychological counseling and hospice care on top of medical care and daily living care. In terms of the scores on the first level indicators, the two batches of pilot cities scored higher on indicators X2, X8, and X9. All samples have a secondary indicator score of 1 for X2. It's can show that when the pilot sites formulate LTCI policies, they are able to clarify the responsibilities of all parties to the policy, implement categorized supervision, and promote in an integrated manner. Analysis of the secondary indicators for X8 reveals that both pilot sites X81, X82 scored 1 point, X83 is slightly lacking. Explain that hospitals and nursing facilities have been set up as places for the provision of long-term care services in all pilot sites. In the future, care places should be gradually expanded to the community to provide more convenient services to insured persons. The mean X9 values for the two batches of pilot cities were 0.78 and 0.8, respectively. Analysis of their secondary indicators revealed that both batches of policy samples scored low on X91. Pilot sites should strengthen financial security by transferring start-up funds for LTCI, establishing a transfer fund or setting up a risk reserve to ensure the smooth implementation of LTCI. Lower scores for X3 in both pilot lots. An analysis of the scores on its secondary indicators shows that localities are at the pilot stage and most of the policies introduced are short-term policies. At the end of the pilot, localities should enact a sustainable LTCI policy to guide the effective implementation of the policy based on the actual situation. We analyzed the policies with the highest PMC scores: P7, P6, P16, and P1, which are the only policies from both batches with a PMC index above 7. All four policies are from the first batch of pilot cities, with an average PMC index of 7.375, exceeding the overall average PMC index of 6.42. Among these, the scores for X2-Policy Function and X8-Service Place in the four pilot cities are all 1, indicating that the policy content in these cities is well-structured, and the issued policies effectively promote the implementation of LTCI. The score of 1 for X8- Service Place indicates that long-term care services in these cities are comprehensive, encompassing hospitals, older adult care institutions, and community service centers. In terms of X4-Insured Object, P6, P7, and P1 achieved full coverage of both urban and rural residents, whereas P16 did not include rural residents in its coverage. The three pilot cities are located in central and eastern China, where the level of economic development is higher than in the northeastern region, leading to more available funding for LTCI implementation and broader coverage. Regarding X65- Welfare Eligibility, only 4 out of the 29 pilot policies included older adult individuals with dementia as insured, including P7 and P1. Qingdao, in Shandong Province, is a key participant in China's LTCI pilot program. Compared to P6 and P16, both located in the eastern region as well, Qingdao benefits from a stronger economic foundation, allowing for more comprehensive welfare benefits. In terms of X7- Nursing Service, P6, P7, and P16 scored 1, as these cities offer comprehensive services to care recipients, including medical care, daily living assistance, preventive care, rehabilitation, psychological counseling, and end-of-life care. P1 didn't receive a full score as it didn't provide preventive care or end-of-life care. In terms of X9- Safeguard Measure, all four policies include clear fund management provisions and have allocated startup funds for LTCI, either by establishing adjustment funds or setting aside risk reserves, ensuring financial support for LTCI implementation. In today's digital era, these cities leverage the internet to promote information sharing for LTCI, facilitating the implementation of LTCI policies.

### 4.2 PMC surface diagram and typical case analysis

**Figure 2:**
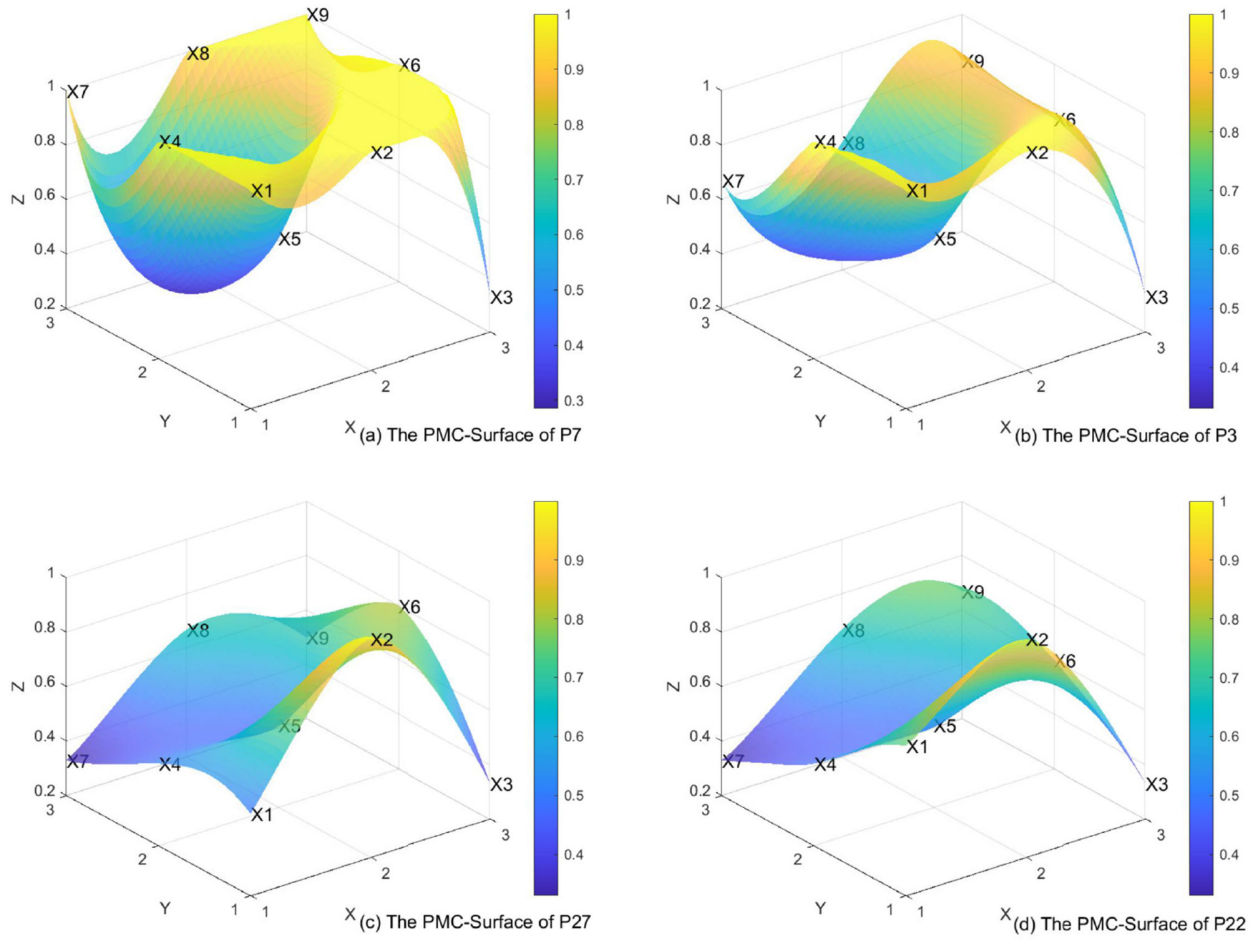
The PMC-surface of **(a)** P7, **(b)** P3, **(c)** P27, and **(d)** P22.

Due to space limitations, the authors selected a few representative policies to illustrate their PMC surface diagrams. We select the highest-scoring policy from the first batch, P7 (Figure 2a), and the highest-scoring policy from the second batch, P3 (Figure 2b), along with the lowest-scoring policies from the first batch, P27 (Figure 2c), and the second batch, P22 (Figure 2d). In the diagram, raised sections indicate higher scores for the first-level variables, while sunken sections indicate lower scores.

P7 ranks first among the policies classified as “Excellent.” As a key city in the first batch of LTCI pilot cities, Qingdao has well-regulated policies and comprehensive functions, achieving universal coverage, offering thorough nursing services, convenient service venues, and robust safeguard measures. Therefore, X1, X2, X4, X6, X7, X8, and X9 all received full scores. However, in terms of funding sources, the policy did not include individual contributions, additional employer payments, or welfare funds, leading to a noticeable dip in the X5 score. Qingdao's policy framework explicitly mandates “the establishment of an urban-rural care service resource allocation mechanism,” resulting in a maximum score (X8 = 1) for this indicator. The implementation of medical insurance fund transfer payments (X55 = 1) further alleviates financial pressures in rural areas, demonstrating an operational pathway for resource mobility across urban and rural jurisdictions. This policy design reflects a systematic approach to addressing spatial disparities in long-term care provision through fiscal redistribution mechanisms and coordinated service delivery networks.

P3 ranks first in the “Excellent” category among the second batch of pilot cities. Kaifeng, as part of the second batch of LTCI pilot cities in China, has well-structured policies and comprehensive functions, benefiting both urban and rural residents. As a result, X1, X2, and X4 appear noticeably elevated in the diagram. However, in terms of funding sources, individual contributions, additional employer payments, and welfare funds were not included, leading to a noticeable dip in the X5 score.

A comprehensive comparison of the two exemplary pilot cities, Qingdao (P7) and Kaifeng (P3), reveals that Qingdao's high score (P7) reflects the comprehensiveness of its policy design. Notably, X7 (nursing services) covers six service categories in Qingdao (P7), demonstrating its effective localization of international experiences (43). In contrast, Kaifeng (P3) received a lower score for X5 (funding sources), highlighting the region's reliance on central government funding due to its underdeveloped economy. Compared to P7 and P3, the surface diagrams of P27 and P22, with lower PMC scores, show more pronounced dips. Nevertheless, the LTCI policy is functionally complete, with high scores in policy regulation, safeguard measures, and service venues, reflected as slight elevations in the surface diagram. There is a need to further expand the funding sources. The coverage of the insured population should be appropriately expanded to include both urban and rural residents, to allow more citizens to access long-term care services.

## 5 Further analysis of policy diffusion based on PMC model

### 5.1 The temporal diffusion exhibits an “M-shaped” curve evolution effect

**Figure 3:**
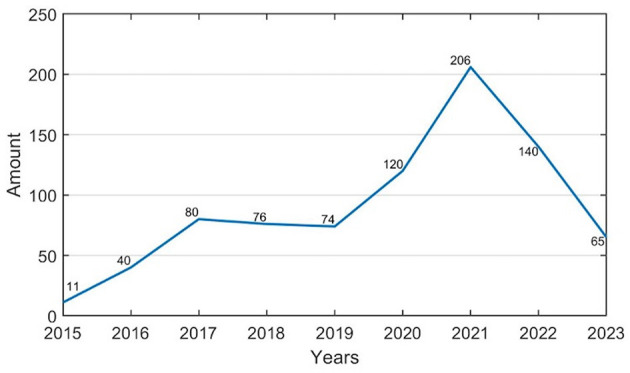
LTCI policies time evolution chart.

The LTCI system in China started relatively late. In December 2006, the State Council issued the “Eleventh Five-Year Plan for Population Development and the 2020 Plan,” proposing to explore the establishment of socialized services such as older adult LTCI. Since then, “LTCI” has appeared multiple times in policy documents. We used the year of publication of LTCI implementation regulations as the horizontal axis and the cumulative number of LTCI systems implemented in cities in that year as the vertical axis to create a line graph (Figure 3).

Based on Figure 3, we can categorize the diffusion of China's LTCI policies over time into three stages. The first stage, before 2016, was the budding pilot stage. This stage was mainly characterized by local, independent exploration. No pilot sites had been identified, and there was no central-level pilot policy announcement, so the number of LTCI policies showed a slow upward trend. Subsequently, between 2017 and 2019, the primary pilot phase was launched. In March 2016, the 13th Five-Year Plan explicitly stated that China would begin exploring the establishment of an LTCI system suited to its national conditions. In June 2016, the Ministry of Human Resources and Social Security issued a policy, marking the first time the LTCI pilot program was explicitly mentioned at the central level. The pilot program was then launched in 15 cities across the country and in two provinces, Shandong and Jilin. The number of LTCI policies introduced at each pilot site in 2017 was nearly eight times that of 2015, marking the first rapid proliferation of China's LTCI policies. This suggests that after the central government clarified the pilot policy, there was a significant response from across the country. Finally, from 2020 to 2023, the pilot phase entered its expansion stage. In May 2020, the National Health Insurance Bureau and the Ministry of Finance issued a policy proposing to expand the pilot scope by adding 14 new pilot cities, bringing the total to 29. In 2021, the number of LTCI policies introduced in each pilot site reached 2.5 times that of 2017, marking the second large-scale proliferation of LTCI policies in China and reaching a peak in the number of introduced policies. Subsequently, the number of policies declined to some extent, indicating that after the central government introduced new policies, localities promptly adjusted previously implemented policies, resulting in an initial increase followed by a decrease.

In the time dimension, China's LTCI policies diffusion follows an “M”-shaped curve, with a slower rise during the period of independent exploration. In both 2016 and 2020, the central government expanded LTCI pilot sites, leading to two periods of rapid diffusion, and the second being more significant. The M-shaped curve reflects the “intervention-response” mechanism inherent in China's policy pilot system. The central government's phased expansion of pilot cities (in 2016 and 2020) directly triggered peaks in policy diffusion, while subsequent local government adjustments (e.g., optimized funding mechanisms in the second batch) contributed to the observed downturns in adoption rates. This pattern challenges Rogers' assumption of “natural permeation” in diffusion processes, instead highlighting the dominant role of administrative forces in shaping policy adoption dynamics (44).

### 5.2 Proximity diffusion effects arising from the interaction of spatial and social proximities

The proximate effects of policy diffusion typically describe how different regions learn from each other based on similarities in certain aspects, ultimately resulting in comparable policy content across regions. When a local policy pilot achieves favorable outcomes, neighboring cities are likely to adopt these policies to achieve similar anticipated effects. The proximity effect is a principal mechanism of public policy spatial diffusion in China. When facing new policy challenges, local governments not only rely on their own past experiences but also draw on the experiences of other cities, especially those in close spatial proximity. The spatial evolution of LTCI in China demonstrates a clear proximity diffusion effect, influenced by both spatial and social proximities.

**Figure 4:**
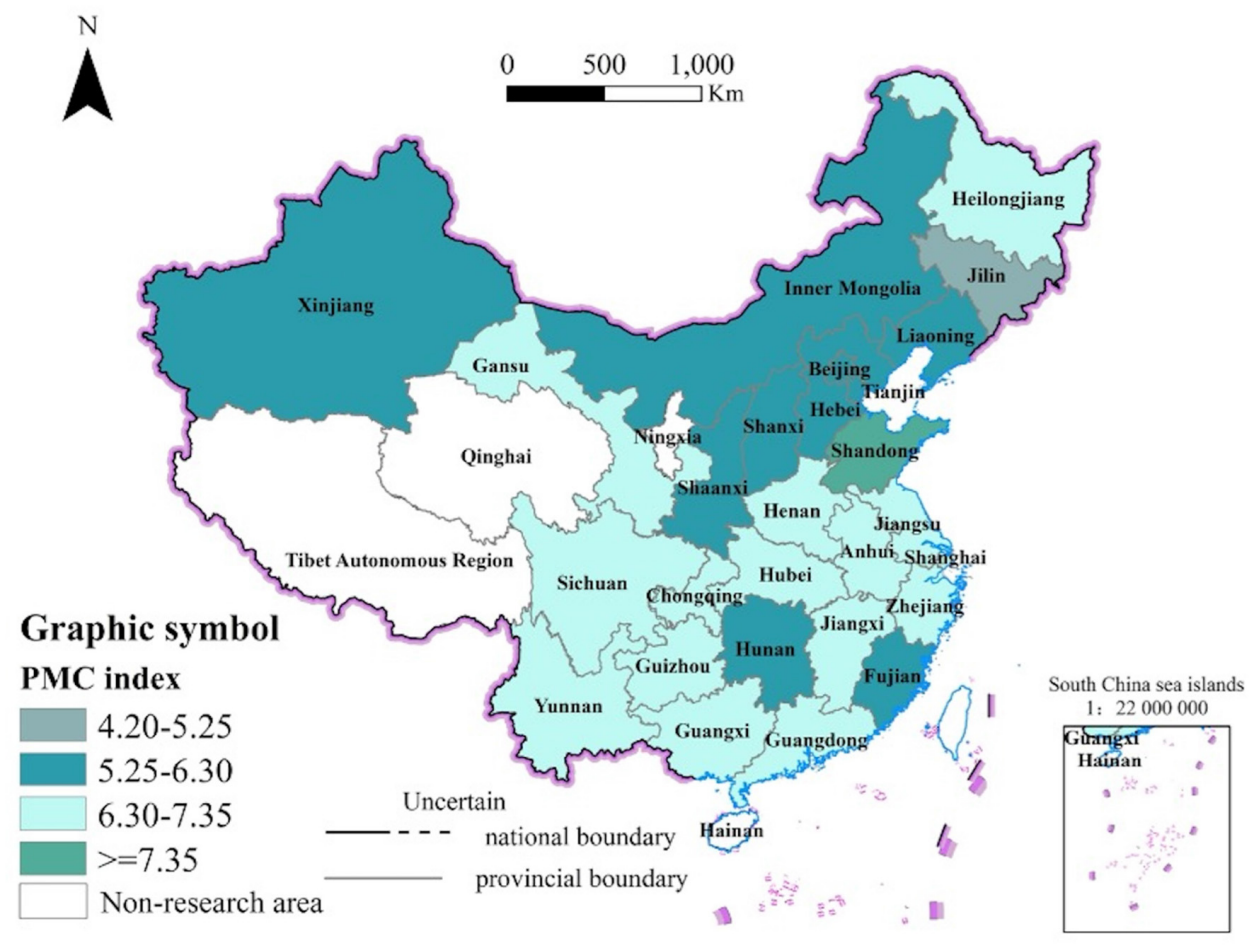
PMC index of LTCI policies in cities.

According to the results calculated from the PMC index, LTCI policies diffusion in China exhibits typical spatial proximity characteristics (Figure 4). Shandong Province, Jilin Province, and the urban agglomerations of the Yangtze River Delta and the Beijing-Tianjin-Hebei region, as the initial pilot areas for the LTCI system, served as diffusion sources, gradually spreading to neighboring cities and demonstrating significant proximity effects. The average PMC index for the middle Yangtze River urban region is 6.67, closely followed by the Central Plains region with an average PMC index of 6.41, and the Yangtze River Delta region with an average PMC index of 6.77. The maximum difference between these indices is only 0.36, highlighting the prominent proximity effect in the policy diffusion process of LTCI. Moreover, regarding X65-Welfare Eligibility, initially only Qingdao included dementia patients as service recipients. Later, Nantong and Shangrao mentioned in their policies that they would gradually expand coverage to include dementia patients based on the implementation of LTCI. In 2023, Suzhou's policy also included dementia patients as eligible for coverage.

The PMC index of four geographically proximate research samples—P1, P12, P9, and P8—is 7.08, 6.96, 6.76, and 6.66, respectively. Based on the primary indicator scores, all four policies received a score of 1 for X2-Policy Function and X8-Service Place, and their scores for X1-Policy Nature, X6-Policy Norm, and X9-Safeguard Measure are largely consistent. When issuing LTCI policies, local governments are often influenced by neighboring cities, adopting a learning approach to formulate their local policies. P7 and P3, which have the highest PMC indexes in the first and second batches, respectively, are also geographically adjacent, with P3 showing clear evidence of policy learning. The two policies were issued in 2021 and 2018, respectively, and P3's 2021 policy scored 1 for X4-Insured Object, covering both urban and rural residents.

Furthermore, policy diffusion characteristics are influenced not only by regional economic development levels but also by local governance structures and administrative capacity, leading to variations in PMC scores. For example, P28 and P2, which rank high in economic development levels, have identical PMC index scores of 6.43, despite not being geographically adjacent. Analyzing the primary indicator scores, P28 and P2 are consistent in X1, X2, X3, X4, X6, and X7, with P2 scoring slightly higher than P28 in X5-Capital Source due to the inclusion of individual contributions. Similarly, P24 and P4, both exhibiting moderate levels of economic development, have PMC index scores of 6.3 and 6.58, respectively, with a difference of 0.28. In regions with lower levels of economic development, such as Gannan Autonomous Prefecture, Qianxinan Buyei and Miao Autonomous Prefecture, the PMC index scores are both 6.38. Based on the primary indicator scores, P24 and P4 are identical in X2, X3, X4, X5, X7, and X8, with the maximum difference between the remaining indicators being only 0.2. This suggests that LTCI policies exhibit a clear social proximity effect during the policy diffusion process. For instance, while cities within the same province–such as Nantong (P8) and Suzhou (P1) in Jiangsu–exhibit identical scores in X8 (service facilities), Suzhou outperforms Nantong in X7 (care services) with a score of 1.0 compared to 0.83. This discrepancy may stem from Suzhou's stronger fiscal support and more efficient administrative coordination mechanisms, underscoring the role of localized institutional factors in shaping policy implementation outcomes.

### 5.3 Hierarchical diffusion effects exhibiting interwoven vertical and horizontal expansion pathways

In a multi-level governmental management system, a vertical hierarchical structure exists between different levels of government. Policy diffusion often follows a top-down trajectory due to an energy differential, resulting in a hierarchical effect on policy dissemination. This differential may include administrative enforcement from the central government toward local governments or policy influence between cities and regions. The diffusion of LTCI policy in China illustrates both the top-down guidance from the central government to local governments and the influence of the eastern regions over the central and western areas.

The hierarchical effects of LTCI policy diffusion in China are mainly reflected in the vertical interactions between the central and local governments. The 2016 policy specifies that the primary funding sources are transfers from pooled medical insurance funds and basic medical insurance for workers. An analysis of secondary variables reveals that both X51-Medical Insurance Pooling and X52- Personal Account in the 29 pilot areas are assigned a value of 1, indicating that the main sources of LTCI funding currently rely on these medical insurance components. However, the 2020 policy emphasizes independent operation, distinct insurance categories, and the independent design and advancement of the LTCI system. Calculations using the PMC index indicate that X53-Individual Payment score for the first batch of pilot cities is 0.4, while for the second batch it is 0.70. For X55-Financial Subsidies, the first batch averages 0.86, with the second batch scoring an average of 0.92. For X56 -Welfare Fund, the first batch averages 0.20, while the second batch scores 0.39. Funding source scores are higher in the second batch of pilot cities, following the 2020 policy toward a more diversified funding approach, reducing reliance on pooled medical insurance funds. This reflects the hierarchical influence of the central government on local governments in guiding LTCI policy diffusion. The central government's policy adopts a “flexible authorization” approach to stimulate local innovation in LTCI system development. The 2020 policy directive mandated independent LTCI framework design, enabling economically advanced regions such as Suzhou (P1) to explore diversified financing mechanisms, while less-developed areas such as Gannan (P29) maintained reliance on central fiscal subsidies. This “categorized authorization” mechanism effectively balances policy uniformity with regional adaptability, creating an institutional environment that accommodates heterogeneous local conditions. The differentiated governance model illustrates how strategic decentralization can maintain national policy coherence while also encouraging context-specific solutions.

Furthermore, the hierarchical effects are also evident in horizontal diffusion among neighboring cities within the same province. These cities often show similarities in their LTCI policy formulations. For example, in Jiangsu Province, both Nantong (P8) and Suzhou (P1) score 1 on the primary indicators X6-Policy Norm and X8-Service Place. This similarity is particularly evident in X65 -Welfare Eligibility, where both cities are among the few pilot sites that include dementia patients as insured subjects, reflecting horizontal imitation in LTCI policy formulation. Similarly, in Xinjiang Province, Urumqi (P24) and Shihezi (P20) both score 1 on the primary indicator X4-Insured Object, with both cities including urban employees and rural-urban residents in their insurance coverage.

## 6 Discussion

Based on the above analysis results, we will further discuss the following aspects. We evaluates both the first and second batch of pilot LTCI policies and performs a comparative analysis with PMC model. The comparison reveals that the overall consistency of LTCI policies in the first batch of pilot cities in China is higher than that in the second batch, with PMC index of 6.61 and 6.23. Among them, X7-Nursing Service policies in the first batch of pilot sites scores 0.16 points higher than the second batch. The score differences of other variables are all within 0.1, indicating a high degree of similarity in policy content between the two batches of pilot LTCI policies in China.

A comparative analysis of multiple indicators reveals that urban governance capacity significantly influences policy diffusion dynamics. The X5 (funding sources) metric reflects regional variations in financial resource structures. Components of X9 (safeguard measures) include “organizational coordination” (X93) and “information management” (X95) scores and “dynamic management” (X96) performance, collectively demonstrate administrative efficiency. The second-batch pilot city of Kaifeng (P3) demonstrated a lower X5 (funding sources) score of 0.5 compared to 0.83 in first-batch cities like Qingdao (P7), highlighting greater fiscal reliance on central government subsidies among less economically developed regions. Concurrently, cities with higher X95 (“information management”) scores under the X9 (safeguard measures) dimension everaged digital governance tools to enhance administrative efficiency such as Suzhou (P1) and Qingdao (P7). Thereby facilitating more effective policy implementation. This disparity underscores the interplay between regional economic capacity and technological adoption in shaping policy execution outcomes.

The PMC indices of policies in both batches of pilot areas are lower with respect to X5-Capital Source and X7-Nursing Service. Currently, in most economically underdeveloped regions of China, long-term care services primarily focus on medical services and daily living care. In contrast, more affluent regions provide a wider range of services, including preventive care, rehabilitation therapy, psychological counseling, and end-of-life care. Despite these offerings, both medical services and daily living care remain inadequate to meet caregiver needs. Given China's vast geographic expanse and the disparities in economic development across regions, differences in the availability and quality of care services are expected. The central government should strengthen top-level design, while local governments must tailor LTCI funding mechanisms and operational methods to local conditions to promote LTCI development in their respective regions. Regarding the X2-Policy Function, X8-Service Place, and X9-Safeguard Measure, the consistency of policies introduced in the two batches of pilot areas is extremely high. The high consistency observed in X2 (policy function) and X9 (safeguard measures) reflects the central government's influence in standardizing local policy frameworks through “guiding opinions,” ensuring that policy functions and safeguard measures align closely with central directives. Central policy documents clearly delineate responsibilities and dynamic management requirements, which local policies must strictly adhere (45). This demonstrates the dominant role of vertical diffusion. However, significant differences are evident across cities in X5 (funding sources) and X7 (nursing services), indicating that governments adjust policies based on fiscal capacity and service needs, selectively adopting neighboring regional policies. This illustrates adaptive learning in horizontal diffusion. For example, eastern cities, such as Qingdao, have incorporated diversified financing models, while western regions, constrained by fiscal limitations, rely solely on medical insurance funds.

Based on the PMC index, we analyze the characteristics of LTCI policies during the diffusion process. This study finds that the diffusion of LTCI policies in China follows an M-shaped curve in time. This study analyzes the characteristics of LTCI policy diffusion using the PMC index. Contrary to the traditional S-shaped curve commonly observed in policy diffusion (46), as noted in the literature, this study identifies an M-shaped curve in the temporal evolution of LTCI policy diffusion in China. The Rogers model primarily focuses on individual-level adoption decisions, whereas the PMC model emphasizes the comprehensiveness and internal consistency of policy texts. While the classical S-curve assumes a single peak in diffusion rates, China's LTCI policy exhibits an M-shaped diffusion pattern, driven by the central government's two large-scale pilot expansions (the first batch of 15 cities in 2016 and the second batch of 14 cities in 2020). By quantitatively assessing policy quality, the PMC model reveals iterative refinements between pilot phases, with the M-curve reflecting the “batch-intervention effect” of policy experimentation. This finding is significant, as some scholars have questioned the universal applicability of the S-shaped model (45). This dynamic complexity, characteristic of China's administratively driven diffusion, cannot be adequately captured by traditional models, highlighting the PMC framework's superior explanatory power in such institutional contexts.

The LTCI policy in China exhibits hierarchical diffusion effects, characterized by both vertical and horizontal diffusion, intertwined with interactions of spatial and social proximity. This includes adjacent spatial diffusion effects and social proximity. Compared to previous studies on policy diffusion, this study also identifies spatial and social proximity phenomena in the diffusion process of China's LTCI policies (47–49). Spatial and social proximity both contribute to the diffusion of China's LTCI policies. The diffusion of LTCI policies in China demonstrates both vertical and horizontal interaction effects (10, 50). Owing to China's distinctive political system, political factors significantly influence policy diffusion (51).

## 7 Conclusion and policy implications

### 7.1 Conclusion

The issue of long-term care for the older adult and those who cannot care for themselves has become an urgent social concern. In response to the increase demand for older adult care, China has been explored LTCI pilot program in 29 cities. However, there is not yet a unified national policy framework. It is crucial and important to research the diffusion characteristics and underlying reasons from the first to the second batch of LTCI policies, which can help establish a unified national analytical framework and enrich the theory of public policy innovation and diffusion in China. This paper utilizes the PMC model to analyze 29 LTCI policies from two batches of pilot cities in China in order to study the characteristics of LTCI policies diffusion.

Designing an idealized policy is a prerequisite for the start of policy implementation and ensure coherence and consistency in policy implementation and execution is also important. Therefore, it is particularly important to study the characteristics of policy content consistency. This paper constructs a new analytical framework for studying policy diffusion. Based on the analysis of policy texts using the PMC model. We focus on the analysis of policy quality and consistency to the diffusion characteristics and exploring the underlying reasons for the diffusion of the LTCI policies. We have expanded the research on the diffusion of LTCI by using the PMC model to construct a multidimensional evaluation index system, providing a systematic evaluation of policy content. This extends LTCI research from traditional qualitative analysis to the quantitative research domain.

The results of PMC model showed that the overall design of China's LTCI policies are relatively reasonable. The PMC index results show that, among the 29 study samples, 25 cities achieved an excellent level, and 4 cities reached an acceptable level, with no substandard policies. This indicates that China's LTCI policies are scientific and rigorous. In a comparison between the two batches, the average PMC index for the first and second batch of study samples was 6.61 and 6.23, respectively. The first batch performed slightly better than the second. Within the two batches, the second batch showed more consistent PMC index, with smaller differences between cities. The selected policy samples in this paper scored well in X1-Policy Nature, X2-Policy Function, X4-Insured Population, X6-Policy Regulations, X8-Service Venues, and X9-Security Measures, demonstrating a high level of consistency. However, the PMC index was relatively low in X5-Funding Sources and X7-Care Services, indicating areas that need improvement.

The policy diffusion analysis based on PMC results reveals the diffusion characteristics of China's LTCI policies across different regions and batches. The diffusion of China's LTCI policies has followed an “M-shaped” curve over time. Overall, it has gone through a process of “local government independent exploration —actions by relevant national departments—spontaneous actions by local governments—initiatives by relevant national institutions—promotion by first batch of pilot cities—national departments summarizing pilot experiences—expansion to more pilot cities.” During the policy diffusion process, there were two large-scale expansions, with the second expansion being larger in scale than the first batch. This is generally consistent with the findings of Li and Kong (10). The present study demonstrates that the PMC model, through its quantitative assessment of policy quality and internal consistency, effectively captures the distinctive M-shaped diffusion pattern of China's LTCI policy. This finding not only extends the explanatory power of traditional policy diffusion theory regarding “administratively-driven diffusion” but also provides methodological insights for studying hierarchical policy experimentation in other national contexts.

Secondly, in terms of spatial diffusion, there is an interaction between spatial proximity and social proximity effects. According to the PMC index, the regional proximity effect of China's LTCI policies is evident, with geographically adjacent areas exhibiting higher policy consistency, demonstrating a clear spatial proximity pattern. At the same time, the economic development levels across regions in China are unbalanced. The PMC index indicates that regions with similar levels of economic development and living standards tend to have higher consistency in their LTCI policies. Therefore, the diffusion of China's LTCI policies also shows a clear social proximity effect. Finally, in terms of diffusion pathways, there is a hierarchical diffusion effect characterized by vertical diffusion intertwined with horizontal diffusion. In China's multi-tiered government management system, there is a vertical hierarchical structure between different levels of government. The diffusion of LTCI policies exhibits a hierarchical effect, divided into the vertical diffusion between the central and local governments and the horizontal diffusion between neighboring local governments. The primary variable scores between the two batches of pilot cities vary, reflecting different focuses in policy formulation.

### 7.2 Policy implications

In view of the above conclusions and combined with the situation of population aging, this paper puts forward the following policy improvement suggestions:

Learning and innovation work together to address practical local challenges. Under the influence of inter-governmental relations with Chinese characteristics, policy diffusion exhibits both convergence and divergence. There are differences in resources, capacities, pressures and needs among local governments, so local governments should not simply learn and copy the policy contents of neighboring regions in the process of horizontal learning. Instead, they should take into account local conditions and formulate LTCI policies that align with their specific circumstances. Local governments should seek innovative breakthroughs in policy learning to solve local practical problems, thereby promoting local economic and social development.

Balancing all aspects of the LTCI policy to promote synergistic policy design that integrates LTCI with pension systems, healthcare, and housing support programs. Balanced consideration of all aspects of the LTCI policy to achieve its comprehensive development. By calculating the PMC index of each pilot site, found that China's LTCI policies are relatively deficient in terms of participants, funding sources, and care services. It is recommended to expand the scope of participants to ensure benefits for the entire population. To broaden the sources of LTCI funding to ensure its sustainable development. To explore the synergy of multiple LTCI stakeholders to provide comprehensive care services. A cross-departmental coordination mechanism should be established to facilitate the creation of centralized “one-stop” service platforms at the national level, effectively bridging LTCI with medical and pension services. Particular attention should be given to comprehensive support for vulnerable populations by incorporating housing subsidy provisions for low-income older adult within LTCI frameworks, thereby enhancing policy inclusivity.

We should seek common ground while respecting differences and promote balanced development in different regions. The imbalance in economic development across regions is a key factor preventing the nationwide harmonization of LTCI policies. The central government might first consider harmonizing policies in regions with similar economic and social conditions before gradually expanding to national harmonization. The promulgation of policies should be differentiated, with regions delineated across the country and unified policies implemented within each region. The goal is to first achieve LTCI policy unification within each region, followed by gradual downward integration, and ultimately the proliferation of LTCI nationwide. While the overall goal is the same, there are variations across regions. Regional integration can effectively mitigate blind imitation among local governments, shorten the policy development gap between regions, and accelerate the nationwide proliferation of LTCI policies.

To achieve differentiated governance support, the central government should establish targeted fiscal transfer payments for less-developed regions while streamlining administrative procedures. Furthermore, performance-based incentive mechanisms should be implemented by incorporating LTCI policy implementation outcomes into local government performance evaluations. This dual approach would promote more equitable policy diffusion across jurisdictions. We should implement regionally differentiated policy designs based on PMC scores, with distinct priorities across geographic regions. Eastern areas should be emphasized service diversification while central and western regions should be prioritized financial sustainability mechanisms. This approach could be operationalized through a “central framework with local flexibility” governance model, which maintains core policy standards while permitting contextual adaptations to enhance regional applicability. Such spatially stratified implementation strategies may optimize policy effectiveness by aligning intervention priorities with regional development characteristics and institutional capacities.

## Data Availability

The raw data supporting the conclusions of this article will be made available by the authors, without undue reservation.
